# Electric Stimulation of Ear Reduces the Effect of Toll-Like Receptor 4 Signaling Pathway on Kainic Acid-Induced Epileptic Seizures in Rats

**DOI:** 10.1155/2018/5407256

**Published:** 2018-02-26

**Authors:** En-Tzu Liao, Yi-Wen Lin, Chun-Ping Huang, Nou-Ying Tang, Ching-Liang Hsieh

**Affiliations:** ^1^Graduate Institute of Chinese Medicine, College of Chinese Medicine, China Medical University, Taichung 40402, Taiwan; ^2^Graduate Institute of Acupuncture Science, College of Chinese Medicine, China Medical University, Taichung 40402, Taiwan; ^3^Research Center for Chinese Medicine and Acupuncture, China Medical University, Taichung 40402, Taiwan; ^4^School of Chinese Medicine, College of Chinese Medicine, China Medical University, Taichung 40402, Taiwan; ^5^Graduate Institute of Integrated Medicine, College of Chinese Medicine, China Medical University, Taichung 40402, Taiwan; ^6^Department of Chinese Medicine, China Medical University Hospital, Taichung 40447, Taiwan

## Abstract

Epilepsy is a common clinical syndrome with recurrent neuronal discharges in the temporal lobe, cerebral cortex, and hippocampus. Clinical antiepileptic medicines are often ineffective or of little benefit in 30% of epileptic patients and usually cause severe side effects. Emerging evidence indicates the crucial role of inflammatory mediators in epilepsy. The current study investigates the role of toll-like receptor 4 (TLR4) and its underlying mechanisms in kainic acid- (KA-) induced epileptic seizures in rats. Experimental KA injection successfully initiated an epileptic seizure accompanied by increased expression of TLR4 in the prefrontal cortex, hippocampus, and somatosensory cortex. In addition, calcium-sensitive phosphorylated Ca^2+^/calmodulin-dependent protein kinase II (pCaMKII*α*) increased after the initiation of the epileptic seizure. Furthermore, downstream-phosphorylated signal-regulated kinase (ERK), c-Jun NH_2_-terminal protein kinase (JNK), and p38 kinase simultaneously increased in these brain areas. Moreover, the transcriptional factor phosphorylated nuclear factor-*κ*B (pNF-*κ*B) increased, suggesting that nucleus transcription was affected. Furthermore, the aforementioned molecules decreased by an electric stimulation (ES) of either 2 Hz or 15 Hz of the ear in the three brain areas. Accordingly, we suggest that ES of the ear can successfully control epileptic seizures by regulating the TLR4 signaling pathway and has a therapeutic benefit in reducing epileptic seizures.

## 1. Introduction

Temporal lobe epilepsy is a neurological disease causing abnormal discharges in the brain, particularly in the cortex and hippocampus. Epilepsy is defined as an abnormal condition of brain imbalance, with unpredictable electrical discharges and seizures. According to clinical studies, 30% of epileptic patients undergo uncontrolled epileptic seizures owing to ineffective antiepileptic drugs. In general, several antiepileptic drugs serve as antiexcitatory or enhanced inhibitory agents to depress seizure occurrence. Accordingly, these medicines have severe side effects on cognition and memory [1, 2]. Epilepsy is the result of an imbalance between excitatory and inhibitory function and can be induced in animal models by overactivation of excitatory neurotransmitter receptors through a kainic acid (KA) injection, which is utilized to initiate epileptic seizures [3]. Another method to induce epileptic seizures involves blocking the inhibitory GABA_A_ receptors, and this can be performed using pilocarpine [4]. Increase of GABA, including gabapentin and sodium valproate, was clinically used for epileptic seizure control with little effectiveness and numerous side effects.

Toll-like receptors (TLR) are mainly located on the cell membrane, but TLR3 and TLR7 are located in the endosomal compartment [5]. TLR2, TLR3, and TLR4 are majorly expressed in microglia, astrocytes, and neurons, and their expression is decreased because of physiological conditions. In pathology-related epileptic seizures, TLRs are increased [6–9]. Activation of TLR2 triggers the signaling pathway for myeloid differentiation factor 88 (MyD88) and further activates nuclear factor kappa-light-chain-enhancer of activated B cells (NF-*κ*B) in the nucleus to increase proinflammatory mediators [10]. Several studies have demonstrated that TLR4 is crucial for regulating inflammatory responses [11–13]. TLR4 is reported to recognize both exogenous pathogen-associated and endogenous damage-associated molecular patterns. TLR4 is also reported to act as a receptor, that is, a marker of microglial activation in central nervous system (CNS) inflammation [14, 15].

Acupuncture has been used in Asia for thousands of years to treat diseases. A number of studies have indicated that electroacupuncture (EA) can be used to treat stroke-induced dementia [16], epilepsy [3], body weight control [17], Parkinson's disease [18], depression [19], and pain [20, 21]. Initially, scientists determined that acupuncture treatment worked through the release of endogenous opiates [22], serotonin [23], and adenosine [24]. The well-known mechanism of acupuncture analgesia involves the release of opiates in CNS [22] and adenosine in the peripheral nervous system (PNS) [24]. A recent study showed that long-term electric stimulation (ES) of the ear significantly reduces inflammatory mediators in CA1 of the hippocampus in KA-induced epileptic seizure in rats [3]. In the current study, we hypothesized that TLR4 and related molecules are crucial for epileptic seizure in rats. ES of the ear is effective in treating KA-induced epilepsy seizure by regulating TLR4 and related pathways in the rat brain.

## 2. Materials and Methods

### 2.1. Animals

Male Sprague-Dawley (SD) rats weighing 200–300 g were purchased from BioLASCO (BioLASCO Taiwan Co., Ltd) and hosted in the animal center of China Medical University (CMU). A 12–12 h light-dark cycle was maintained, and the room temperature was controlled at 25°C. Adequate food and water were provided. The Animal Care and Use Committee of CMU approved the use of these animals. In addition, all procedures were performed according to the* Guide for the Use of Laboratory Animals* (National Academy Press).

### 2.2. Epileptic Seizure Rat Model

Thirty SD rats were placed in a stereotaxic apparatus in a prone position under isoflurane (Aerrane, Canada) anesthesia administered through a vaporizing system (MATRX VIP 3000, Midmark, USA). Hair from the rats' scalp was cut using surgical scissors, and a surgical knife was used to incise the scalp at the midline to expose the skull. Stainless steel screw electrodes, which were placed on the dura above the bilateral sensorimotor cortices, served as recording electrodes. A reference electrode was placed at the frontal sinus for electroencephalogram (EEG) recordings. Bipolar electrical wires were passed through the subcutaneous tissue and around the neck muscles for electromyogram (EMG) recordings. The electrodes were plugged into a conductor, which was fixed to the skull with dental acrylic cement. These electrodes were then connected to EEG- and EMG-monitoring machines (MPIOOWSW, BIOPAC Systems, Inc., CA, USA). Epileptic seizure behaviors were confirmed using a video-recording epileptic behavioral analysis system (SeizureScan, Clever Sys., Inc., Virginia, USA), and both EEG and EMG findings were recorded during a conscious and free-moving state for at least 4 days after electrode implantation. On EEG recordings, intraperitoneal injection (i.p.) of KA (12 mg/kg) was observed to mainly induce epileptic seizure behaviors, namely, wet-dog behavior, facial myoclonia, paw tremors, and epileptiform discharges. The epileptic seizures were confirmed on the observation of behavioral changes, including wet-dog shakes, paw tremors, and facial myoclonia in a freely moving and conscious state and on that of epileptiform discharges based on EEG recordings. Rats exhibiting more than 250 wet-dog shakes and more than 100 facial myoclonia plus paw tremors were selected. Epileptic seizure behaviors were observed on EEG and EMG recordings at 15 min before and 3 h after the KA injection. We followed the methods of Liao et al., 2017 [3].

### 2.3. Grouping

The rats were randomly divided into five experimental groups, and each group contained six rats as follows: (1) control group, in which the rats were peritoneally injected with phosphate buffer solution (PBS); (2) KA group, in which the rats were injected with KA (12 mg/kg i.p.); (3) 2 Hz ES group, in which the rats received 2 Hz ES (using clip electrodes, with the cathode at the ear apex and anode at the ear lobe; stimulus frequency: 2 Hz; stimulus intensity: visual ear twitch; stimulus duration: 20 min/day, with each ear receiving the stimulus for 10 min alternately); (4) 15 Hz ES group, in which the rats received 15 Hz ES (using clip electrodes, with the cathode placed at the ear apex and anode at the ear lobe; stimulus frequency: 15 Hz; stimulus intensity: visual ear twitch; stimulus duration: 20 min/day, with each ear receiving the stimulus for 10 min alternately); (5) sham group, in which the clip electrodes were connected to an electric stimulator without electric charge. ES was applied for 3 days per week for 20 min/day for 3 weeks, starting from the day following the KA injection. All the rats were sacrificed at 3 weeks, and their brains were removed.

### 2.4. Western Blot Analysis

Following brain extraction, the frontal cortex, hippocampus, and somatosensory cortex were immediately excised for protein extraction. The total protein was prepared by homogenizing the hippocampi for 1 h at 4°C in a lysis buffer containing 20 mmol/L of imidazole: HCl (pH 6.8), 100 mmol/L of KCl, 2 mmol/L of MgCl_2_, 20 mmol/L of ethyleneglycoltetraacetic acid (pH 7.0), 300 mmol/L of sucrose, 1 mmol/L of NaF, 1 mmol/L of sodium vanadate, 1 mmol/L of sodium molybdate, 0.2% Triton X-100, and a proteinase inhibitor cocktail. From each sample, 30 *μ*g protein was extracted and analyzed through a bicinchoninic acid protein assay. The protein was subjected to 10%–15% sodium dodecyl sulfate-tris-glycine gel electrophoresis and was transferred to a nitrocellulose membrane. The membrane was blocked with 5% nonfat milk in TBST buffer (10 mmol/L of Tris, pH 7.5; 100 mmol/L of NaCl; and 0.1% Tween 20) and incubated overnight at 4°C with the primary antibodies (anti-TLR4, anti-pCaMKII*α*, anti-pERK, anti-pp38, anti-pJNK, and anti-pNF*κ*B) in TBST containing bovine serum albumin. Peroxidase-conjugated antibody (1 : 500) was used as the secondary antibody. The membrane was assessed using the ECL-Plus protein detection kit. We followed the methods of Liao et al., 2017 [3].

### 2.5. IHC Staining

The rats were anesthetized with isoflurane and then intracardially perfused with saline. The brains were removed and postfixed in the same fixative overnight at 4°C. After briefly washing with PBS, the brains were transferred to a 30% sucrose solution in 0.01 M PBS for cryoprotection, and coronal sections containing the hippocampal area were cut into 16 *μ*m thick slices through cryosectioning. The sections were preincubated for 10 min at room temperature with 10% normal goat serum in PBS to avoid nonspecific binding. The sections were incubated overnight at 4°C in PBS containing the primary antibodies (anti-TLR4, anti-pERK, and anti-pNF*κ*B). The sections were subsequently incubated with the biotinylated-conjugated secondary antibody (diluted at 1 : 200; Vector, Burlingame, CA 94010, USA) for 10 min at room temperature, followed by incubation with avidin-horseradish peroxidase complex (ABC kit, Genemed, USA). The sections were then visualized using 3,3′-diaminobenzidine as a chromogen. During the incubation steps, the sections were washed with PBS thrice for 10 min per cycle. The stained hippocampus slices were sealed under coverslips and then examined for the presence of immune-positive hippocampal neurons using a microscope (Olympus, BX-51, Japan) with a 40x numerical aperture (NA = 1.4) objective. We followed the methods of Liao et al., 2017 [3].

### 2.6. Statistical Analysis

All data are presented as mean ± standard deviation. Statistical significance among the control, KA, 2 Hz ES, 15 Hz ES, and sham groups was analyzed through one-way ANOVA, followed by Tukey's post hoc test. A *p* value of <0.05 was considered statistically significant.

## 3. Results

### 3.1. Effect of Ear ES on the Levels of TLR4 in Prefrontal Cortex of KA-Induced Epileptic Seizure Rats

**Figure 1:**
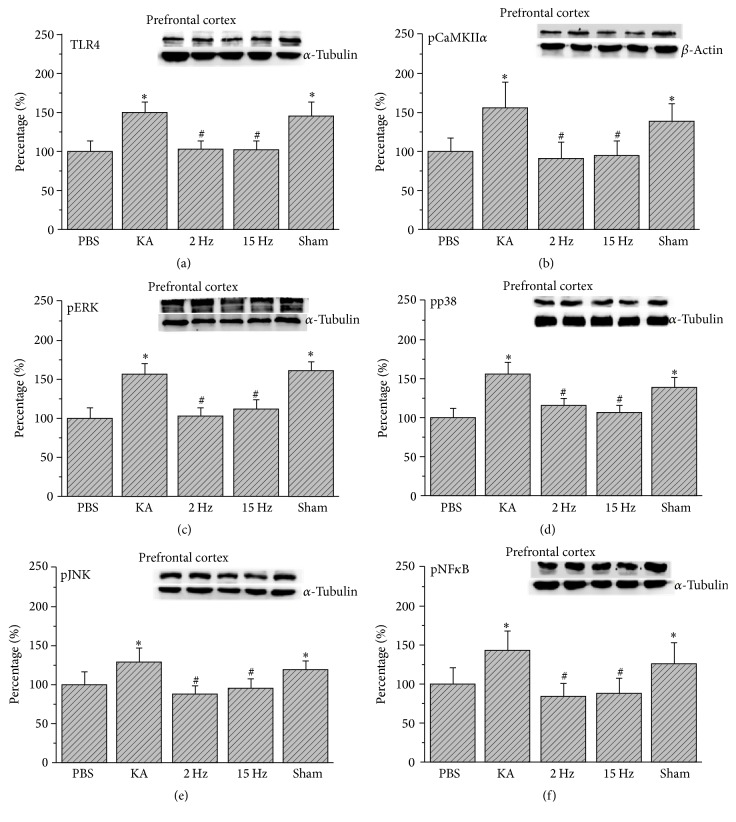
Expression levels of TLR4 and related molecular proteins in the prefrontal cortex. (a) TLR4, (b) pCaMKII*α*, (c) pERK, (d) pp38, (e) pJNK, and (f) pNF*κ*B expression levels in the prefrontal cortex of the PBS, KA, 2 Hz, 15 Hz, and sham ES groups (from left to right). PBS = phosphate-buffered saline; KA = kainic acid-induced epileptic rats; 2 Hz ES = 2 Hz electrical stimulation of the ear; 15 Hz ES = 15 Hz electrical stimulation of the ear; sham = sham-operated electrical stimulation of the ear. ^*∗*^*p* < 0.05 compared with the control group. ^#^*p* < 0.05 compared with the KA group. The western blot bands at the top indicate the target protein. The lower bands correspond to the internal controls (*β*-actin or *α*-tubulin).

We first used the western blot technique to investigate TLR4 and related signaling pathways in the frontal cortex. Our results indicated that TLR4 increased in KA-induced epileptic rat prefrontal cortex (Figure 1(a), 149.67%  ±  14.16%, *p* < 0.05, *n* = 6). The potentiation was reversed by either 2 Hz ES (Figure 1(a), 103.35%  ±  10.29%, *p* < 0.05, *n* = 6) or 15 Hz ES (Figure 1(a), 102.72%  ±  10.27%, *p* < 0.05, *n* = 6). However, we did not observe this phenomenon in sham controls (Figure 1(a), 146.18%  ±  17.45%, *p* > 0.05, *n* = 6). TLR4 activation can further initiate Ca^2+^ influx into cell plasma. We then checked the Ca^2+^-mediated second messenger pathway in rat prefrontal cortex. We showed that pCaMKII*α* significantly increased by KA injection (Figure 1(b), 156.28%  ±  31.97%, *p* < 0.05, *n* = 6). This phenomenon was reversed by 2 Hz ES (Figure 1(b), 90.62%  ±  21.13%, *p* < 0.05, *n* = 6) and 15 Hz ES (Figure 1(b), 95.41%  ±  17.53%, *p* < 0.05, *n* = 6), except in sham controls (Figure 1(b), 139.26%  ±  21.73%, *p* > 0.05, *n* = 6). We also checked the serial downstream molecules such as the MAPK subfamily pERK, pp38, and pJNK. We found that immunopositive signals of pERK, pp38, and pJNK were increased in the KA group (Figures 1(c)–1(e), 157.52%  ±  13.28%, 155.22%  ±  16.59%, 129.59%  ±  17.04%, *p* < 0.05, *n* = 6). All the results were reversed by 2 Hz ES (Figures 1(c)–1(e), 102.15%  ±  10.21%, 115.73%  ±  10.02%, 88.63%  ±  8.99%, *p* < 0.05, *n* = 6) and 15 Hz ES (Figures 1(c)–1(e), 112.02%  ±  12.77%, 107.51%  ±  7.86%, 96.16%  ±  10.35%, *p* < 0.05, *n* = 6), except in sham controls (Figures 1(c)–1(e), 160.89%  ±  12.76%, 139.36%  ±  12.96%, 119.05%  ±  12.01%, *p* > 0.05, *n* = 6). To investigate the transcriptional factor, we examined the level of nucleus factor pNF*κ*B. Our data demonstrated that pNF*κ*B increased in the prefrontal cortex of epileptic rats (Figure 1(f), 143.81%  ±  25.04%, *p* < 0.05, *n* = 6). These results were reversed by 2 Hz ES (Figure 1(f), 84.61%  ±  16.78%, *p* < 0.05, *n* = 6) or 15 Hz ES (Figure 1(f), 87.59%  ±  19.7%, *p* < 0.05, *n* = 6), except in sham controls (Figure 1(f), 126.4%  ±  26.34%, *p* > 0.05, *n* = 6).

### 3.2. Effect of Ear ES on the Levels of TLR4 in Hippocampus of KA-Induced Epileptic Seizure Rats

**Figure 2:**
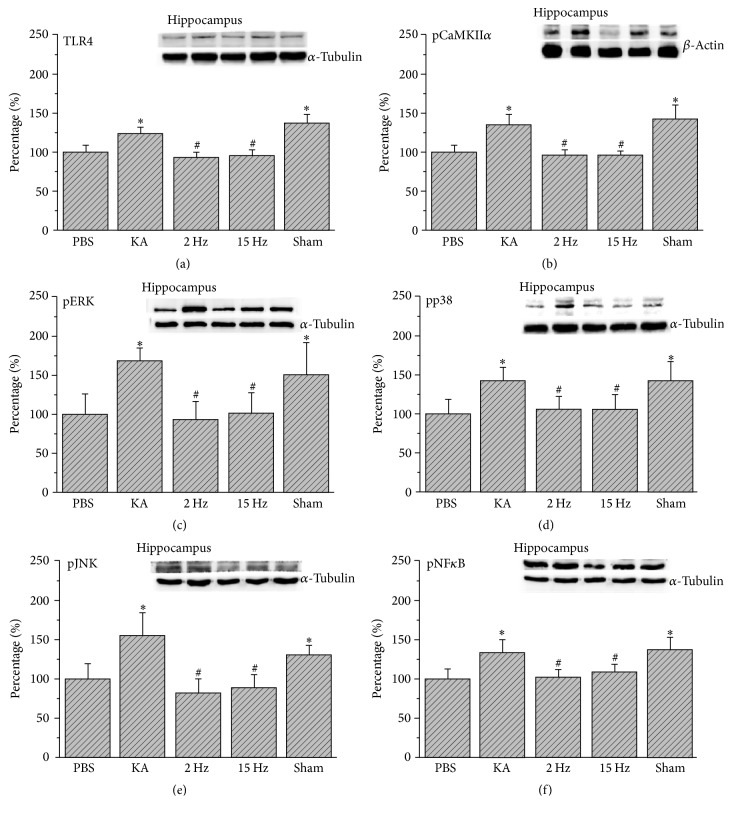
Expression levels of TLR4 and related molecular proteins in the hippocampus. (a) TLR4, (b) pCaMKII*α*, (c) pERK, (d) pp38, (e) pJNK, and (f) pNF*κ*B expression levels in the hippocampus of the PBS, KA, 2 Hz, 15 Hz, and sham ES groups (from left to right). PBS = phosphate-buffered saline; KA = kainic acid-induced epileptic rats; 2 Hz ES = 2 Hz electrical stimulation of the ear; 15 Hz ES = 15 Hz electrical stimulation of the ear; sham = sham-operated electrical stimulation of the ear. ^*∗*^*p* < 0.05 compared with the control group. ^#^*p* < 0.05 compared with the KA group. The western blot bands at the top indicate the target protein. The lower bands correspond to the internal controls (*β*-actin or *α*-tubulin).

We then showed that TLR4 increased in the hippocampus of KA-induced epileptic rats (Figure 2(a), 124.45%  ±  7.25%, *p* < 0.05, *n* = 6). This result was reversed by 2 Hz ES (Figure 2(a), 92.76%  ±  7.39%, *p* < 0.05, *n* = 6) and 15 Hz ES (Figure 2(a), 95.91%  ±  7.75%, *p* < 0.05, *n* = 6), except in sham controls (Figure 2(a), 137.24%  ±  10.54%, *p* > 0.05, *n* = 6). We also showed that pCaMKII*α* further increased by KA injection (Figure 2(b), 135.53%  ±  12.53%, *p* < 0.05, *n* = 6) and decreased by 2 Hz ES (Figure 2(b), 95.27%  ±  7.63%, *p* < 0.05, *n* = 6) and 15 Hz ES (Figure 2(b), 94.76%  ±  7.17%, *p* < 0.05, *n* = 6), except in sham controls (Figure 2(b), 142.36%  ±  18.66%, *p* > 0.05, *n* = 6). In addition, serial downstream molecules, such as pERK, pp38, and pJNK, significantly increased in the KA group (Figures 2(c)–2(e), 167.18%  ±  37.36%, 140.88%  ±  17.74%, 155.81%  ±  28.37%, *p* < 0.05, *n* = 6). These results were then attenuated by 2 Hz ES (Figures 2(c)–2(e), 95.07%  ±  21.24%, 105.32%  ±  17.18%, 83.93%  ±  17.58%, *p* < 0.05, *n* = 6) or 15 Hz ES (Figures 2(c)–2(e), 100.52%  ±  26.75%, 105.36%  ±  19.71%, 89.31%  ±  16.45%, *p* < 0.05, *n* = 6), except in sham controls (Figures 2(c)–2(e), 150.55%  ±  21.37%, 140.69%  ±  25.59%, 131.58%  ±  12.07%, *p* > 0.05, *n* = 6). Furthermore, we demonstrated that pNF*κ*B increased in the hippocampus of epileptic rats (Figure 2(f), 134.59%  ±  15.12%, *p* < 0.05, *n* = 6). The potentiation was attenuated by both 2 Hz ES (Figure 2(f), 102.94%  ±  10.27%, *p* < 0.05, *n* = 6) and 15 Hz ES (Figure 2(f), 109.48%  ±  9.42%, *p* < 0.05, *n* = 6), except in sham controls (Figure 2(f), 138.48%  ±  16.27%, *p* > 0.05, *n* = 6).

### 3.3. Effect of Ear ES on the Levels of TLR4 in Somatosensory Cortex of KA-Induced Epileptic Seizure Rats

**Figure 3:**
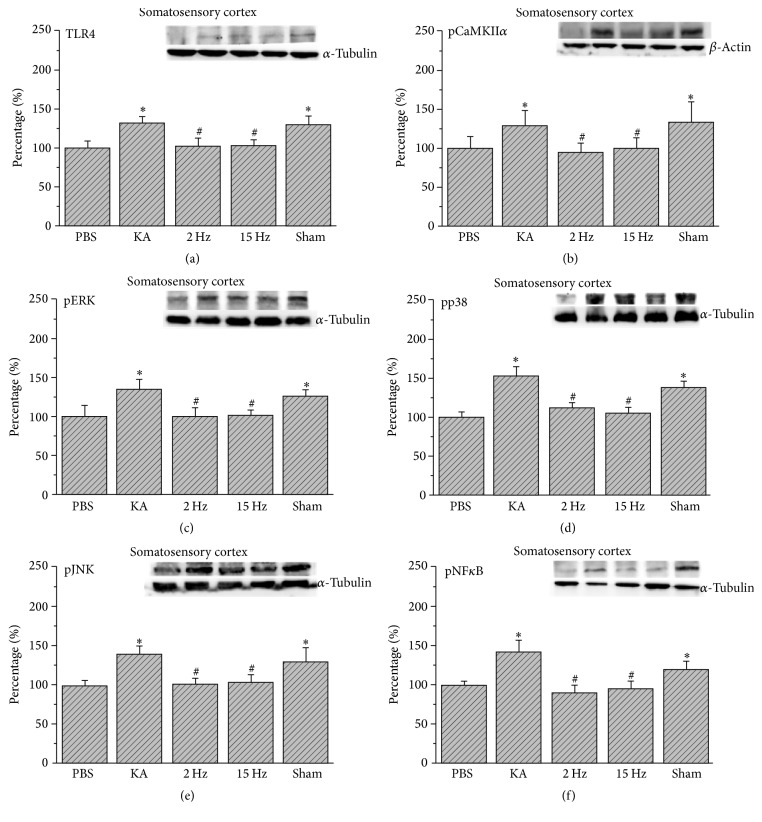
Expression levels of TLR4 and related molecular proteins in the somatosensory cortex. (a) TLR4, (b) pCaMKII*α*, (c) pERK, (d) pp38, (e) pJNK, and (f) pNF*κ*B expression levels in the somatosensory cortex of the PBS, KA, 2 Hz, 15 Hz, and sham ES groups (from left to right). PBS = phosphate-buffered saline; KA = kainic acid-induced epileptic rats; 2 Hz ES = 2 Hz electrical stimulation of the ear; 15 Hz ES = 15 Hz electrical stimulation of the ear; sham = sham-operated electrical stimulation of the ear. ^*∗*^*p* < 0.05 compared with the control group. ^#^*p* < 0.05 compared with the KA group. The western blot bands at the top indicate the target protein. The lower bands correspond to the internal controls (*β*-actin or *α*-tubulin).

We further checked if the TLR4 signaling pathway was involved in the somatosensory cortex and found that TLR4 increased in the somatosensory cortex of the epileptic rats (Figure 3(a), 132.07%  ±  8.91%, *p* < 0.05, *n* = 6). This phenomenon was reversed by 2 Hz ES (Figure 3(a), 102.42%  ±  10.69%, *p* < 0.05, *n* = 6) and 15 Hz ES (Figure 3(a), 103.57%  ±  7.79%, *p* < 0.05, *n* = 6), except in sham controls (Figure 3(a), 129.75%  ±  10.02%, *p* > 0.05, *n* = 6). We further determined that pCaMKII*α* increased in epileptic rats (Figure 3(b), 129.45%  ±  17.92%, *p* < 0.05, *n* = 6), and this increase was reduced by both 2 Hz ES (Figure 3(b), 94.55%  ±  12.38%, *p* < 0.05, *n* = 6) and 15 Hz ES (Figure 3(b), 98.77%  ±  14.92%, *p* < 0.05, *n* = 6), except in sham controls (Figure 3(b), 134.73%  ±  25.33%, *p* > 0.05, *n* = 6). Similarly, pERK, pp38, and pJNK also increased in the KA group (Figures 3(c)–3(e), 134.69%  ±  13.49%, 153.43%  ±  12.26%, 139.17%  ±  11.44%, *p* < 0.05, *n* = 6), and this increase was reversed by both 2 Hz ES (Figures 3(c)–3(e), 100.69%  ±  10.09%, 112.83%  ±  6.03%, 102.01%  ±  7.47%, *p* < 0.05, *n* = 6) and 15 Hz ES (Figures 3(c)–3(e), 101.32%  ±  7.82%, 105.35%  ±  8.18%, 103.92%  ±  9.62%, *p* < 0.05, *n* = 6), except in sham controls (Figures 3(c)–3(e), 126.57%  ±  8.52%, 138.17%  ±  8.02%, 129.19 ± 18.78%, *p* > 0.05, *n* = 6). Furthermore, we determined that pNF*κ*B increased in the somatosensory cortex of epileptic rats (Figure 3(f), 143.11%  ±  14.24%, *p* < 0.05, *n* = 6), and this potentiation was attenuated by both 2 Hz ES (Figure 3(f), 91.13%  ±  8.55%, *p* < 0.05, *n* = 6) and 15 Hz ES (Figure 3(f), 95.16%  ±  9.84%, *p* < 0.05, *n* = 6), except in sham controls (Figure 3(f), 121.05 ± 10.12%, *p* > 0.05, *n* = 6).

### 3.4. Effect of Ear ES on Immunohistochemistry Analysis of TLR4-pERK-pNF*κ*B Expression in Hippocampus and Somatosensory Cortex

**Figure 4:**
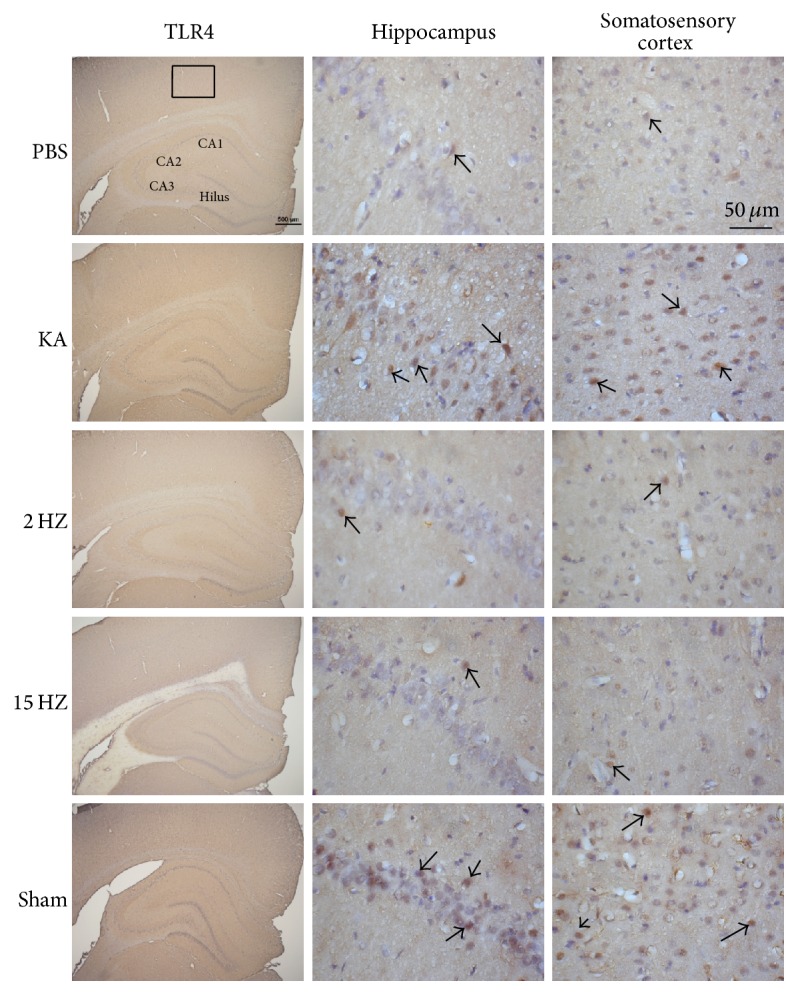
Expressions of TLR4 in the hippocampus and somatosensory cortex of PBS, KA, 2 Hz ES, 15 Hz ES, and sham rats. Arrows identify immunopositive neurons.

**Figure 5:**
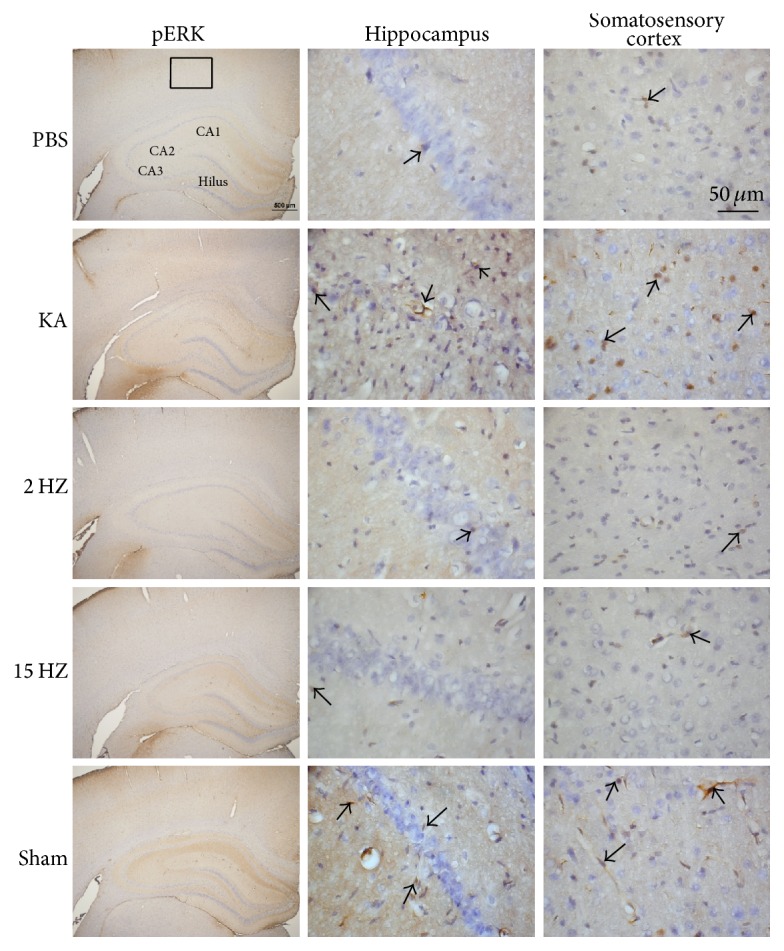
Expressions of pERK in the hippocampus and somatosensory cortex of PBS, KA, 2 Hz ES, 15 Hz ES, and sham rats. Arrows identify immunopositive neurons.

**Figure 6:**
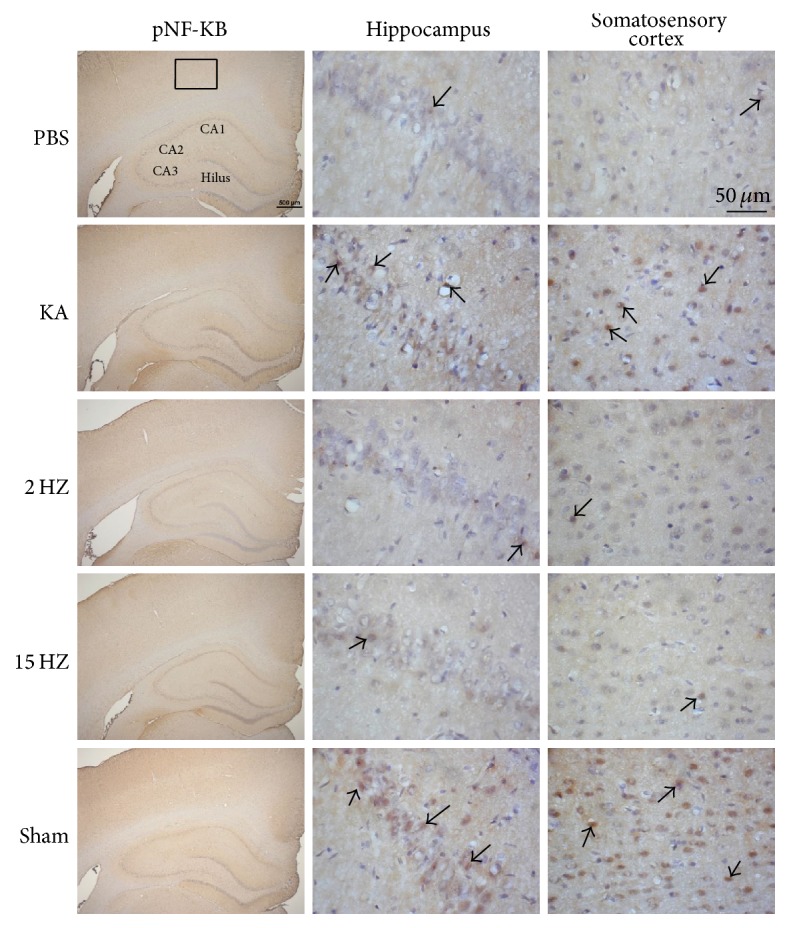
Expressions of pNF*κ*B in the hippocampus and somatosensory cortex of PBS, KA, 2 Hz ES, 15 Hz ES, and sham rats. Arrows identify immunopositive neurons.

**Figure 7:**
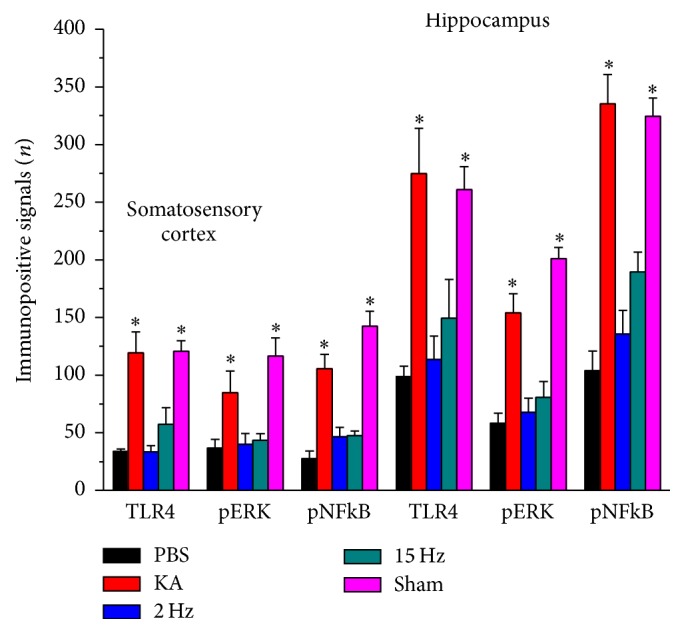
Immunopositive signals of TLR4, pERK, and pNF*κ*B in the hippocampus and somatosensory cortex of PBS, KA, 2 Hz ES, 15 Hz ES, and sham rats. The immunopositive signals of TLR4, pERK, and pNF*κ*B from hippocampus and somatosensory cortex were presented in each group. ^*∗*^*p* < 0.05, as compared to PBS group.

Immunohistochemical staining, visualized in brown color, demonstrated that TLR4 expression level was expressed in control rat hippocampus and somatosensory cortex, which further increased in KA-induced epileptic rats. The overexpression of TLR4 was reversed by both 2 Hz ES and 15 Hz ES, except in sham controls (Figure 4). A similar pattern was observed in pERK expression; we found that pERK increased after KA injection, and this was reversed by both 2 Hz ES and 15 Hz ES, except in sham controls (Figure 5). We further determined that pNF*κ*B also increased in the KA group, and this potentiation was reversed by both 2 Hz ES and 15 Hz ES, except in sham controls (Figure 6). All data were analyzed and plotted as Figure 7.

## 4. Discussion

The current study provides information about the molecular effects of ES on TLR4-related mechanisms in KA-induced epileptic rats. Several articles have suggested that brain inflammation is an intrinsic feature of epileptic seizures [25]. In inflammatory conditions of CNS, TLR can be activated by inflammatory mediators such as interleukin-1 (IL-1) and high-mobility group box-1 (HMGB1). Activation of TLR further triggers a signaling pathway comprising cAMP, NF*κ*B, and COX-2 [25]. Song et al. reported that both the mRNA and protein expression of TLR4 in rat hippocampus were reliably increased with pentylenetetrazole injection [26]. They suggested that TLR4 contributes to epilepsy and may also contribute to epileptic therapy [26]. Luan et al. reported that HMGB1 was detected in the cytoplasm of astrocytes in the cortex, suggesting that it is released by glial cells [27]. Levels of HMGB1 and its receptors were increased in inflammatory conditions of the cortex. In brain inflammation, stronger TLR was observed in gray matter [28]. A recent article reported that activation of TLR4, which is determined by agonist injection, influences neuronal excitability to recruit sterile inflammatory responses [29]. We previously suggested that COX-2 levels in the hippocampus and the number of COX-2 immunoreactive cells in the hippocampal CA1 region increased after KA-induced epileptic seizures, and this increase was reduced through a 6-week application of ES at the ear or EA at the ST36-ST37 acupoints [3]. This has an anti-inflammatory effect, which can reduce COX-2 overexpression, suggesting that ES and EA are beneficial for the treatment of epileptic seizures [3]. Our results indicated that 2 Hz or 15 Hz ES successfully controlled epileptic seizures by regulating the TLR4 signaling pathway.

Han reported that there are four endogenous opioids, namely, *β*-endorphin, enkephalin, endomorphin, and dynorphin that can be released by EA in the brain [22]. They indicated that *β*-endorphin is released mainly around the periaqueductal gray matter at 2 Hz and 15 Hz EA. In addition, enkephalin and endomorphin increased majorly in the spinal cord by 2 Hz EA. Furthermore, dynorphin is released at the spinal cord level but only under high frequency EA. EA is suggested to be involved in the inflammatory response through the hypothalamus-pituitary-adrenal axis and the nervous system [30]. EA is also indicated to reduce the inflammation-induced expression of neurokinin-1 in the spinal cord dorsal horn (SCDH) of rats [31]. Activation of opioid receptors markedly reduces peripheral inflammation in local tissues [32] and suppresses Ca^2+^ currents in primary afferent neurons [33]. Several reports suggest that TLR4 plays a crucial role in epilepsy [27, 29, 34]. Accordingly, we suggest that ES increases the level of opiates in both peripheral and central levels to reduce TLR4 expression through inhibition of inflammatory responses. In Chinese medicine, ES or EA is usually used to treat epilepsy. Auricular sensory afferents constituted by vagus, glossopharyngeal, and facial nerves can contribute to parasympathetic nerve activity [35–37]. Vagus nerve stimulation has been used to reduce the occurrence of seizures in pediatric patients with intractable epilepsy [37, 38]. ES has also been reported to activate parasympathetic tone to reduce epilepsy [38]. Recently, acupuncture is reported to reduce hippocampal neuronal death through inhibition of inflammatory mediators in epileptic KA-induced mice [3]. Our recent study reported that pERK1/2 expression was increased in the KA-induced seizure rats and can be further reduced by both auricular and somatic EA at 6 weeks after induction [39]. Li et al. also indicated that spontaneous seizures and associated ERK activation could contribute to the proliferation in epilepsy model [40]. In addition, recent article showed that NF*κ*B signaling pathway is crucial in a spike-wave discharges (SWD) characterizing absence epilepsy in WAG/Rij rats [41]. We suggested that 2 Hz or 15 Hz ES successfully controlled epileptic seizures by attenuating the pERK and pNF*κ*B signaling pathway.

In conclusion, ES treatment leads to a decrease in inflammatory TLR4 expression in the prefrontal cortex, hippocampus, and cerebral cortex of epileptic rats. Furthermore, pCaMKII*α*, pERK, pp38, and pJNK are involved in this inflammatory process. Moreover, transcriptional pNK*κ*B levels in the three brain areas also were altered by 2 or 15 Hz ES. Our data strongly demonstrate an anti-inflammatory effect of ES in KA-induced epileptic rats. Thus, the knowledge obtained here supports its use in the treatment of epilepsy.
